# Abstracts and Gleanings

**Published:** 1887-11

**Authors:** 


### ABSTRACTS AND GLEANINGS.

**Oil of Erigiron.**—Dr. Bartholow (*Medical Age*) says: I am in a position to reaffirm the statements formerly made. and to express my belief that in oil of erigeron we have a valuable remedv for certain cases of menorrhagia and metrorrhagia and, probably, passive hemorrhages in general. In some cases of neurasthenia it now and then happens that menorrhagia becomes an embarrassing complication; but I have found the erigeron oil to act very efficiently, and whilst it has moderated the flow to proper dimensions, has improved the general condition and lessened the nervous irritability.

For the most part the use of the oil of erigeron for the conditions above mentioned, are not novel. Finding, however, that it is a really effective remedy in menorrhagia and metrorrhagia, I then had recourse to it in these conditions having their origin in albuminuria, and then it was I ascertained that it checked remarkably the waste of albumen. From this point it was but a step to observe how far this remedy affects the course of Bright's disease —or such forms of albuminuria as are due to tubal and interstitial nephritis and cases combining these two morbid processes. No attempt was made to arrive at an exact differentiation, if indeed such be possible. It suffices to state that the chronic cases of nephritis united by the common symptom—albuminuria were included. In the chronic form of Bright's disease, erigeron oil lessens the amount of albumen, lowers the abnormal vascular tension, and improves the general condition. It also seems to have a most favorable action on the headache, the nausea and other symptoms of uræmic character. Whether it be actually curative in cases not too far advanced, can be determined only by careful and sufficiently protracted trials. Thus far it can be said to promise well.

Oil of erigeron also acts in a very beneficial manner in cystitis, lessening the irritability of the bladder and diminishing the catarrh. In a case of prostatic disease with very great irritability of the bladder and much catarrh it seems to have an excellent effect.

In catarrh of the bronchitis and in cough of a nervous character, I have seen very considerable relief given by it. Indeed, in these affections it has seemed to me to afford as much relief as *terebene*, which has been much vaunted of late and with justice.

In administering it, I have usually directed it in 5-drop doses every three or four hours. It may be conveniently exhibited dropped on a lump of sugar. It appears to me to do better in moderate doses rather frequently administered, to maintain a constant impression, than in occasional l[illegible]e doses.

Besides the foregoing practic[illegible] experience it may be mentioned that oil of erigeron is antiseptic and in this capacity may prove useful. But if it had no other practical applcations than those suggested by my own experience, it will deserve a prominent position as a remedy.—*Medical Age.*

**Diagnosis and Treatment of Typhoid Fever.**—I may say at the outset in regard to diagnosis of typhoid fever that there are few cases which do not vary markedly from the type described in the books. I begin the treatment with almost the same assurance of success (and that in two weeks at the farthest) as in ordinary ague.

When called early and when the exact grade of the fever is not really determinable, I first give three or four small doses of calomel followed by a free dose of castor oil. When the case is established two grains of powdered blue mass. combined with as much ipecac as the stomach will bear, every few hours while there is a tendency to constipation.

When the constipation is replaced by diarrhæa Dover's powder is substituted for the ipecac ; the amount being dependent on the condition of the bowels. Half way between the powders I give a teaspoonful of the following emulsion :

Oil turpentine, three drams ; compound spirits of lavender, half an once; laudanum, twenty minims ; sugar and gum arabic each two ounces ; water enough to make six ounces.

At bed time all medicine is stopped ; the patient is given a warm sponge bath ; the bowels are bathed in turpentine. The patient is then put to bed and no more medicine given until after the next breakfast. The diet is carefully watched and as much cold water given as the patient wishes. All special indications are of course met. About the eighth or twelfth day the tongue usually becomes moist and the coating very heavy and loose, and seemingly ready to be scraped off. Calomel in one, two or three grain doses is then given, followed in a few hours by castol oil. The tongue then usually clears up, the temperature approximates normal, the patient begins to call for food, and convalescence is established. Tonics and mineral acids then take the place of calomel. During the several years I have followed this treatment, I have had but one patient to visit later than the fourteenth day, and he recovered the third week. I rarely have a patient unable to sit up in a chair to rest every day throughout the entire sickness. The abnominal distention, bowel hemorrhages, high temperature, cough, confused delirium, deafness, bed-sores and abscesses, I formerly had in my typhoid fever cases in common with so many others, no longer appear since I have adopted the mild mercurial treatment. Those who have failed with the calomel treatment, have, in opinion, given it too early in large doses, discontinued it too soon, and have used tonics before they were indicated by the breaking of the fever. Tonics always do harm if given before the fever is broken, and this particularly the case with quinine, which, given properly, is the tonic in typhoid fever. —Dr. W. O'NEAL MENDENHALL, *in Medical Standard.*

**Red Wine in "Bed Wetting."**—In 1840 a professor under whom I studied in Baden, stated that the physician of an orphan and fondling asylum where children were kept till they reached their fourteeth year, cured night wetting of beds by red wine in two weeks. The wine was given at bed time and in the morning before eating. Fifteen years ago a 62-year old man found himself unable to retain his urine more than five minutes. Recalling the red wine cure to mind I gave him some home made red wine (half blackberry, half elderberry), and he held his water for fifteen minutes and then returned for a second dose. Subsequently he was able to hold his water for two hours during a jury trial. I subsequently cured the urinary incontinence of a fourteen-year-old girl with it, and later that of the other children. In one case it is said to have failed, but as the child's guardian was a fanatical spiritualistic phohibitionist, it is doubtful whether the child ever received the remedy. Two old men, one over sixty, one seventy-six, were cured by it. The dose given was for children one to four drams, and for adults three ounces. The dose given at the orphan asylum was double this of pure wine (port) unobtainable here. A mixture of blackberry and elderberry wine is an excellent substitute. The remedy deserves a trial, as it is certainly palatable.—H. M., in *Standard*.

**Uses of Menthol.**—Menthol is most commonly used in the form of pencils; its solution or ointments, although possibly not so convenient, are probably more lasting in their effects.

Macdonald used a ten-per cent. alcoholic solution. Steward used a solution in bromethyl. The following is a formula for a useful ointment:

℞. Mentholi.................................part i,
Ol. olivari.................................part ss,
Lanolini.................................parts viiiss.
F. unguentum.

Sig.—Lanolin migraine ointment.

For many uses, as applications to the mucous membranes, the following formula, which gives a semi-fluid product, is useful; the substance resulting may be conveniently applied by a camel's hair pencil:

℞. Mentholi.................................part j,
Dissolve in ol. olivari.........................parts iij,
And add lanolini.........................parts vi,
F. unguentum.

In the treatment of burns the following lotion may be used in place of ointments·

℞. Mentholi.................................part j,
Dissolved in ol. olivari.........................parts ix,
And add aq. calcis.........................parts x.

Menthol may also be used in combination in plasters, and has in this form manifold applications.

For toothache from caries a small crystal of menthol may be placed in the hollow tooth, or a mixture of equal parts menthol and chloral hydrate. As is the case when camphor and chloral are mixed, a fluid results. A pledget of cotton may be saturated with menthol and chloral and placed in the hollow of the tooth.

Rabon recommends menthol as a snuff in the treatment of coryza. The effect of menthol as a local anæsthetic is much weaker than its analgesic effect. The proposal of Rosenberg to combine menthol with cocaine for the production of local anæsthesia in the nose and pharynx cannot be considered of great value. In the treatment of reflex nuroses, whose origin was the nasal cavity, Rosenburg obtained excellent results with small bougies of glycerin and gelatin which contained one-sixth grain of menthol; these were inserted into the site of the lesion.—*Therapeutic Gazette.*

**Report of the English Commission on Pasteur's Hydrophobia Inoculation.**—The English Commission appointed in April, 1886, consisting of James Paget, Sir Joseph Lister, Sir Henry Roscoe, Sir Richard Quain, Dr. Lauder Brunton, Prof. Burdon-Sanderson, Dr. George Fleming, and Mr. Victor Horsley, has just presented its report to Parliament. This report, which is based upon observations made by certain members of the committee who visited M. Pasteur, and upon experiments performed by the secretary, Mr. Horsley treats upon all the questions at issue and is a powerful defense of M. Pasteur's method.

With regard to the first claim of M. Pasteur, that his inoculations were capable of protecting persons from the risk of infection if bitten by a rabid dog, the committee reports, "it may be deemed certain that M. Pasteur has discovered a method of protection from rabies comparable with that which vaccination affords against small-pox."

As to the second claim, M. Pasteur has discovered "a treatment capable of preventing the development of the disease in persons bitten by rabid dogs," after considering all the observations of M. Pasteur and performing a number of experiments to test the same, the committee fully endorses it.

The commission has proved that the virus of hydrophobia, as claimed by M. Pasteur, is located in the medulla and spinal cord of the affected animal; that inoculation of this virus in healthy animals, dogs and rabbits, is, as well as the bite of rabid animals, capable of producing the disease, the only difference being that in the former case the stage of incubation is shorter; that this virus is attenuated by exposure of the organs in dry air; and, finally, that inoculation performed with this attenuated virus is capable of preventing the development of hydrophobia, provided this protective inoculation be begun during the period of incubation. The committee estimates not less than one hundred lives have been saved from October, 1885, to December, 1886, by M. Pasteur's treatment.

With regard to the stand taken by some of M. Pasteur's critics that the inoculation and not the bite were the cause of the development of hydrophobia in some of the patients treated, the committee considers that there is no evidence to sustain such a position.—*New Orleans Medical Journal.*

**Thorough and Complete Antisepsis**, says our special correspondent, is now almost entirely used in the New York hospitals, though some operators entertain only the idea of cleanliness, ignoring altogether the views of Tyndall and his sea of floating micrococci. One of the most ardent supporters of antisepsis is Dr. Lewis S. Pitcher. professor of Clinical Surgery in the New York Post-Graduate school. A few hours before resorting to any operation, the patient is thoroughly washed and scrubbed, and the part to be operated upon is well wrapped in bichloride rollers; a pugative is also administered and a very light meal allowed. When the hour arrives for the operation, ether is the anæsthetic generally administered, and always by one well practised in the art. Towels wrung out in hot water and then immersed in bichloride solution, 1 to 2,000, are now laid around the patient. The point of incision and all adjacent points are wiped over with a solution of iodoform and ether. The operator is dressed in a long linen frock something like a "linen duster." He and his assistants wash their hands and arms to the elbows, first, in hot water, and then in bichloride, 1 to 2,000. The incision is douched with the bichloride of the same strength as the blood accumulates. No sponges are used—cheese-cloth soaked in the sublimate solution taking their place. Drainage is effected by drainage tubes or catgut ligatures, either one of which must have been soaked for two months in some antiseptic oil. The wound having been closed, it is covered with sublimated sawdust bags about four inches square, or larger, if necessary; these likewise having been subjected to the same antiseptic process. Over the sawdust bags are now placed several layers of antiseptic cheese-cloth (when the wound happens on an extremity the cheese-cloth is applied in the form of a roller), and, finally, a cotton bandage over the forgoing completes the dressing. All instruments are immersed in a solution of carbolic acid, 1 to 30 or 40, and, as an additional precaution, an antisptic spray is constantly kept up in the room.—*Practice.*

**Chloroform and Ether.**—The relative merits ot these two anæsthetics were recently discussed by the New York Academy of Medicine. Dr. A. G. Gester opened the discussion, and stated that the administration of ether is always, and under all circumsiances, safer than chloroform. The false cry that ether is entirely free from danger is responsible for the reckless way it is used in hospitals to-day. Chloroform in the hands of a well-trained and conscientious anæesthetizer, he believes just as safe as ether. There is only one intrinsic counter-indication to its use—a fatty and weak heart; while there are three to ether—fat or weak heart, kidney and lung trouble. He is of the opinion that *valvular lesions* of the heart are not in themselves a contra-indication to chloroform; but that a *weak* heart, from whatever cause, be it fatty degeneration or great anæmania, or from nervous influences, is the only valid objection to the use of this anæsthetic. He lays particular stress on the danger of attending functional heart-weakness from nervous influences, as fright or excessive fear, it may be, of small operations.

Dr. H. Knapp uses ether as the universal anæsthetic and considers it safer than and just as manageable as chloroform.

Dr. Weir inclines to ether and rejects the dictum that kidney disease contra-indicates its use.

Dr. W. Gill Wylie agrees, in the main, with Dr. Gerster, except that he prefers to give ether because it is safer.

Dr. John A. Wyeth's views may be formulated as follows: Chloroform must be preferred in almost all operations in children under *six* years of age, and in women during childbirth. Chloroform may be chosen in preference to ether when ether has been tried without producing anæasthesia. Chloroform may be used when ether cannot be obtained. The existence of nephrit s would be the only reason for changing these conclusion.

Dr. Munde believes ether the safer of the two when properly administered. In hospitals it was generally left to those least qualified to perform what he considers one of the most important duties connected with an operation. For short operations he prefers chloroform, and also in obstetrics, except when he wishes to stimulate uterine contractions in tedious labor.—*Practice.*

**Physiological Action of Cocaine.**—Dr. Freiberg, of Kowno, Russia, has arrived at the following conclusions respecting the physiological action of cocaine (*Berl. Klin. Woch.*, No. 10). Cocaine is a powerful anæsthetic, its action being limited to the surface in its immediate neighborhood; and when brought into contact with an exposed nerve the anæsthesia is distributed over the periphery, but the central end of the nerve and motility are unaffected. In doses of $\frac{1}{2}$ grain to 1 grain in rabbits, and rather more in dogs, cocaine causes anæsthesia of the cornea, dilatation of pupils, retraction of eyelids, and exophthalmos; tonic and clonic spasms may occur, with loss of consciousness, and often fatal issue. The epileptiform spasms are doubtless of cortical origin. A difference as to the anæsthesia is observed between dogs and rabbits when cocaine is applied to nerve trunks. In dogs reflexes are abolished; in rabbits they are increased. Cocaine has a special affinity for the sensory and terminal sensory fibres of the cortex cerebri, whose function it inhibits. The disturbance of co-ordination may be attributed to interrupted conduction in the sensory fibres, both central and peripheral; and the convulsions, which are the main cause of death in cocaine poisoning, are due to the vasomotor spasm and anæmia of the cortex. Bromide of potassium and the application of cold to the surface counteract this tendency; but with the first appearance of symptoms of cerebral anæmia, these toxic effects of cocaine may be mitigated or banished by nitrite of amyl.—*Pacific Record.*

**Creosote in Phthisis.**—Dr. J. Solis-Chohen (*Polyclinic*) has used beechwood creosote in half minim doses daily for several years in phthisis to retard decomposition of undigested nutriment in stomach and bowels. It is given in a gelatine capsule, with powdered extract of licorice to give it bulk, and, according as the indications may demand, powdered digitalis, quinine, iron, iodoform, or other drugs required, added. The formula most frequently used by Dr. Cohen is: Powdered iodoform, 30 grains; pure creosote (beechwood), 15 minims ; powdered extract of licorice, sufficient to make a mass, which is divided equally into 30 parts, which are dispensed in gelatin capsules, of which one is taken three times a day, after meals. Additional capsules containing one grain of iodoform are supplied when it is desired to increase the quantity of that drug without increasing the amount of creosote. For the last twelve years he has rarely found it necessary to prescribe cod-liver or other restoratives.—*Standard.*

**Tartar Emetic in Labor.**—Dr. Caleb Green, Homer, N. Y., (*Philadelphia Medical Times*), says that tartar emetic is one of the most efficient agents in promoting parturition. When the pulse is tense, the os rigid, the skin dry and hot, the advance of the head slow, antimony in small doses produces a most prompt and happy change for the better. The pulse softens, the skin moistens, the rigid os relaxes, the vigina becomes bathed with mucus, and the uterine contractions hasten on to a speedy termination of the labor. It is not a substitute for ergot, but it has properties which make it much oftener available as a means of hastening labor to a happy termination. If the dose can be graduted so as to produce its effects as a parturient just short of nausea, and especially short of vomiting, the effect seems to be better than where vomiting occurs.—*Standard.*

**The Treatment of Dysentery.**—During a service of thirty-five years in the British army in India, Mac Dowell had valuable experience with dysentery and treatment, and declares that its horrors are vanishing since the following treatment has been employed: 1. An adult takes a dose of at least twenty to twenty-five grains or ipecacuanha. 2. The stomach is prepared to receive this dose by the patient's taking an hour beforehand, twenty drops of tincture of opium, and having a mustard plaster put over his stomach. The ipecacuanha is given in the form of large pills, taken in the evening before going to bed, never in the morning, or in the course of the day. Subsequently, nothing ought to be drank. This treatment is continued every evening, and in this way by the third morning blood, mucous, and pain have generally disappeared. During the day bismuth is allowable.

Small doses of ipecac are only harmful: they were used for two hundred years in India without removing the mortality.—*Allgemeine med. Central Zeitung*, June 29, 1887.

**Dental Anæsthesia**—M. Georges Vian claims to have solved the problem of local anæsthesia in dentistry. After numerous trials of solutions of different strengths, he has found that the soft parts about the maxillæ may be rendered completely insensible by the use of cocaine, associated with a two per cent. solution of carbolic acid. Five minutes before operating, M. Vian dissolves five centigrams (one grain) of hydrochlorate of cocaine in fifty centigrams (ten drops) of the solution, and injects it into the gums, half-way between the neck and the extremity of the root of the tooth. Half of the solution is injected on the palatine, and the remainder on the labial side, pressure being made by the finger, when the needle is withdrawn, to prevent the exit of the fluid. Anæsthesia is perfect in three minutes. The quantity of cocaine advised by M. Vian seems somewhat large, but it is said to have been so used in eighty-seven cases, without causing any unpleasant sympt ms.—*Medical and Surgical Reporter.*

**Salicylate of Sodium.**—We quote from the *London Practitioner* as follows: The action of drugs in megrim and gout is remarkably similar. Trousseau and others have used colchicum with benefit in megrim, and other observers have remarked on the similar curative effects that certain purgatives, as calomel, have in both gout and megrim; and, again, others have used iod. pot. with considerable success; but the great value of salicylate of sodium in some of these headaches is more remarkable; it seems to me to be most certainly curative and not merely palliative, as it removes the concomitant gastro-intestinal troubles along with the headache. Thus a dose of brom. pot. and sp. ammon. aromat. will sometimes remove a slight headache, but it will probably return; with salicylate treatment it is quite a different matter. the headache is gone once and for all, and shows no sign of return for a considerable period; its action in this respect is very similar to that of calomel, it seems to free the secretions of the mouth, and, at the same time, slightly relaxes the bowels.

The dose of salicylate, as recommended by Dr. Brunton, is two to three or four doses or more, and begun when the headache first comes on: this is sufficient.—*Medical Times.*

**The Value of Lantanin in Malaria.**—Lantanin is an alkaloid, extracted by Negrete from *Yerba sagrata*, of the family of verbenas. Buiza noticed first the quinine-like action of the drug on the circulation, and also found it to slow nutrition and to lower the temperature. Intermittent fevers that prove refractory to quinine have yielded to the influence of 30 grains of lantanin. The most delicate stomachs tolerate the drug. In order to produce antipyretic effects in febrile conditions, the dose employed is from 15 to 30 grains in twenty-four hours, given in pill form. In malaria the drug is administered immediately upon the paroxysm. Ninety-five times out of a hundred no further paroxysms will appear.—*Lancet.*

**Extraction of a Splinter of Iron from the Vitreous Humor by Means of an Electro-Magnet.**—On April 20, 1887, a blacksmith's apprentice was admitted to the clinic of Dr. Louis Wolfberg (Breslau) suffering from a splinter of iron which penetrated the right eye at the lower and inner part of the margin of the cornea. It produced a wound about two millimeters long. Ophthalmoscopic examination revealed the presence of a shining piece of metal lying in the vitreous humor. The splinter could be seen to move when a magnet was placed near it while the patient was lying still. Dr. Wolfberg applied the magnet of Voltolini to the outer surface of the lid and bulb for three days (April 23 to 26)

without success. On April 26 he introduced, through the wound made by the splinter, a cataract knife of Arlt, passing it one centimetre into the eye-ball and making the external wound about eight millimeters long. The point of the magnet was applied to the wound and the splinter of iron quickly appeared at the opening, whence it was withdrawn with forceps. The splinter was three millimeters long and half as broad. The patient recovered entirely.—*Klin. Monatsbl. fur Augenheilkunde.*

**"The Treatment of Ulcus Cruris by a New and Successful Method of Dr. Unna."**—The method is as follows: After the leg has been carefully cleaned and shaven to the full extent of the ulcer, the latter is powdered over with iodoform or subnitrate of bismuth or boracic acid, or any other similar pulverized antiseptic. It is then covered with a layer of cotton wool and the healthy skin painted with warm zinc glue, prepared with five parts of oxide of zinc, three of glycerine, six of water and five of gelatine by weight. While the glue is still warm and moist on the leg a (carbolized) gauze roller (Lister) is wound firmly around the whole in such a manner as to cause the traction to work in the direction from healthy skin to the center of the sore. The dressing remains unchanged for three days to a week.

He has had complete success in this manner with twenty-five cases of ulcus cruris, including varicose ulcers, callous, eczematous, inflamed, weak ulcers, one sloughing one and three healthy ones. The dressing relieves pain, allows the patient to use the leg as if it were well, compresses without causing any retention of the discharge, and produces healing in a comparatively short time.

An exact copy of the ulcers thus treated was taken at the commencement by a plan of his own, viz : a piece of fine cambric was placed closely on the ulcer and thoroughly wetted. The outlines of the sore, as well as of dermatitis, eczema, pigmentation, etc., surrounding it, are all perfectly perceptible through this wet covering, and are easily traced with an aniline pencil. The cambric is then spread out to dry, and the tracing transferred to paper. A series of these tracings were shown to the section, and the application of the dressing also practically demonstrated.

The dressing, when dry, forms a firm compact casting. It is easily cut open with a pair of knobbed knee scissors, and then peels off rapidly.—*Correspondent N. O Medical Journal*

**Antifebrin.**—At a recent meeting of the Societe Vaudoise de Medicine, Dr. Paul Demieville, of Lausanne, read a paper on Antifebrin as a Nervine Remedy. He had used the drug in eleven cases of sciatica, eight of lumbago, seven of intercostal and mammary neuralgia, eight of trigeminal neuralgia, eleven of headache (megrim, dyspeptic, and chlorotic cephalalgia, etc.), three of the forearm and hand (probably identical with "ulnar neuralgia," described by Dr. McNaught, of Rossendale, in the *Journal* of April 30th, 1887, page 933), seven of painful menstruation, two of senile gangrene, three of the tabes, six of epilepsy, cancer, nettlerash, hepatic colic, etc. The remedy was given (in adults) as a rule in half-gramme doses, from one to four times daily; if that failed, three-fourths of a gramme, and even one gramme doses were given once, twice, or thrice a day. On the whole, Dr. Demieville is satisfied that antifebrin is an anodyne. In painful affections the drug scarcely ever fails to give marked relief, the pain disappearing within a period varying from a quarter of an hour to two hours. Its sedative action is very often accompanied by a decided hypnotic effect, which is extremely welcome in cases in which pain is associated with obstinate sleeplessness. In a certain proportion of cases the anodyne effects are only temporary. In other cases relief lasts for a more or less prolonged space of time, a new attack yielding to another course of the drug. But in many cases a complete cure is effected within a couple of days or so. As an anodyne, antifebrin is recommended by the author especially for the relief of the agonizing pain of senile gangrene and cancer. The drug seems to diminish the frequency of fits in epilepsy; at any rate, it had this effect in five out of six cases in which it was tried by Dr. Demieville. He recommends it also in hysterical fits and in infantile convulsions. When used for a considerable period, as, for instance, in incurable cases of organic disease, the drug loses its effect somewhat in time; on being discontinued, however, for some days, it seems to recover its activity. With regard to secondary effects, Dr. Demieville states that he has never known it to produce rigors or noises in the ears; but he has sometimes observed diaphoresis and slight giddiness, and once (in a drunken epileptic, aged fifty-one) delirum and hallucinations, resembling those of salicylism. As a rule, the drug is well borne by healthy digestive organs, but in certain cases of gastric disturbance it may give rise to temporary loss of appetite and dyspepsia, more rarely to nausea and vomiting, and possibly to diarrhœa. In one case lachrymation and a pricking sensation about the eyes were complained of after several one gramme doses had been taken.—*British Medical Journal for August.*

**Marriage by Syphilitics—When Safe.**—Dr. Morrow (*Journal Cutaneous and Venereal Diseases*, April 1887,) in a paper on the duration of the syphilogenic capacity in relation to marriage, presents the following conclusions:

1. The facts of every-day observation show that there is nothing constant in contagion, nothing certain in heredity. Many men marry with a syphilis in full activity of secondary manifestations, and never infect their wives or transmit the disease to their offspring.

2. The modern division of syphilis into secondary and tertiary periods based upon anatomical forms and processes does not furnish a safe criterion for determining the contagious or non-contagious cha acter of the lesions.

3. The completion of the second stage does not always mark the definite disappearance of the virulent principle; clinical experience shows that late lesions are exceptionally, but none the less certainly, the source of contagion.

4. The contagi us activity of syphilis and its susceptibility of hereditary transmi-sion cease after the third or fourth year as a rule, yet observations show that the qualities sometimes continue in force much longer and may be manifest in the fifth and sixth year, and even later.

5. The aptitude of syphilitic parents to procreate diseased children may persist after the cessation of all specific manifestations.

6 The precise date when the syphilitic organism reaches the limit of its contagious or transmissive power does not admit of mathematical expression.

7. This limit probably varies in different cases, and many circumstances contribute to adv ance or defer it.

8. The type of the syphilis, the constitutional peculiarities of the patient, the character of the treatment, the presence or absence of certain conditions which are recognized as factors of gravity in syphilis, all exert a gratifying influence.

9. All these elements should be considered in deciding upon the advi-ability of a syphilitic man to marriage. Each case must be studied upon its individual merits.

10. The direct paternal transmission of syphilis, without preliminary infection of the mother, may be classed among the most conclusively established facts of medical science.

11. It is, therefore, a dangerous doctrine to teach that the sole risks a syphilitic man introduces into marriage consists in the contagious accidents he may bear upon his own person.

12. The arbitrary designation of a limit of three, or at most, four years, as perfectly safe for a syphilitic man to marry, with or without treatment and irrespective of the actual existence of specific lesions, is unwaraanted by science or the teachings of experience.—*American Lancet.*

**Stenocarpine the New Substitute for Cocaine.**—A short time since Dr. Seward described an alkaloid he had discovered which had properties very similar to cocaine. He accidentally found it in the leaves of a tree growing in Louisiana, called the "Tear Blanket Tree." Dr. Knapp obtained some of this alkaloid from Dr. Seward and experimented with it sufficiently to reach the following results. In general it has the same properties as cocaine, differing mainly in that it has greater powers as a mydriatic.

Hence, in all cases in which the iris is inflamed stenocarpine is to be preferred to cocaine. When there is a tendency to glaucoma in cases of iritis it is to be preferred to atropine. If we desire anæsthesia without mydriasis it is inferior to cocaine. Hence cocaine is better in most operative procedures. The paralvsis of accommodation by stenocarpine only lasts half as long as atropine, but longer than hæmotropine. Hence for ophthalmoscopic uses it is preferable

It has no more effect upon the unbroken cutis than has cocaine. Small doses injected under the skin produce the same toxic effects as cocaine. Large doses produce alarming general symptoms; violent tetanic convulsions, opisthotonos, dilation of the pupils, excessive acceleration of the pulse and respiration and prostration.

Introduced into the veins stenocarpine produces almost instant death by arrest of respiration and pulsation. It is dangerons to inject even small quantities into any vascular tissues.

The symptoms of poisoning are similar to those of strychnia. It would seem then that the use of this alkaloid was confined to the treatment of cases in which the iris was involved, especially if there was a tendency to glaucoma. Perhaps farther observation will determine farther ends that it will attain better than other remedies.—*American Lancet.*

**A New Era in Cataract Extraction.**—For sometime past Dr. Julian J. Chisholm, of Baltimore, has, in the after-treatment of cases of cataract extraction, dispensed with all bandages and restraint as well as with darkened rooms. He operates under cocaine anæsthesia, closes the operated eye and fixes the lid with a piece of adhesive plaster The other eye is allowed to remain open and the patient is not even confined to his bed, the sunlight only being excluded from his room. He is permitted to walk about, talk and eat his meals, as usual.

Dr. Chisholm has operated for cataract ninety-eight times since May, 1886, and followed this plan of after-treatment, losing but a single eye, and that from septic infection. A number of other prominent occulists have adopted this plan and have expressed their satisfaction with it.

If the results hereinafter continue as good, cataract extraction will be robbed of all its terrors, for patients, subjected to the orthodox padding and bandaging of both eyes, the dorsal decubitus, restraint of the hands and darkened rooms, look on their confinement as a hideous nightmare, and many prefer to use their single redeemed eye rather than to undergo a second period of torture. —*Medical Review.*

**"Take My Advice,** and when you get a prescription put up at a drug store never ask how much it is," said one gentleman to another, the other day. "Why not?" he was asked. "Because the clerk will 'size you up,' as the boys say, guess how much money you have got and charge you your pile." "What do you advise?" Just this. When the urbane compounder of the medicines hands forth your prescription, just look wise and lay down a quarter. Now the chances are that the drugs in the preparation don't cost over a dime. He will look at the quarter, study a minute, and then make up his mind that he has been foolish enough to sell you the same dose for twenty-five cents at some past time, and he'll take it and not say a word. Lay down a dollar, however, and it will be just the same--Try it.—*Boston Medical and Surgical Journal.*

**A Test for Arsenic in Wall-Paper.**—A simple and easily applied test for arsenic in wall-paper has been devised by Mr. F. F. Grenstted. No apparatus is needed beyond an ordinary gas-jet, which is turned down to quite a pin-point, until the flame is wholly blue; when this has been done, a strip of the paper suspected to contain arsenic is cut one sixteenth of an inch wide and an inch or two long. Directly the edge of this paper is brought into contact with the outer edge of the gas flame a gray coloration, due to arsenic, will be seen in the same. The paper is burned a little, and the fumes that are given off will be found to have a strong garlic-like odor, due to the vapor of arsenic acid. Take the paper away from the flame and look at the charred end—the carbon will be colored a bronze-red; this is copper reduced by the carbon; being now away from the flame in a state of division, the copper is slightly oxidized by the air, and on placing the charred end a second time not too far into the flame, the flame will now be colored green by the copper. By this simple means it is possible to form an opinion without apparatus, and without leaving the room, as to whether any wall-paper contains arsenic, for copper arseniate is commonly used in coloring wall-papers.—*British Medical Journal, December 11th, 1886.*

**Method of Inducing Respiration.**—Dr. Enos Blackwell reports a method of resuscitating the new-born infant in an asphyxiated condition. It has one very decided merit, that of immediate application, but is, as Dr. Blackwell says, a procedure which embodies the principles of the Marshall Hall method. The child is laid on the palms of the accoucheur's hands, and then rapidly tossed with a quick motion, this being done while the placenta is still attached. The rapid movement makes the arms fly up, thus lifting the chest walls, and the infant takes air with a sudden sob. Although a rough and ready method, the author has found it highly successful in many instances.—*Weekly Medical Review.*

**Whitlow.**—Dr. Strong, (Ontario Medical Association) after denouncing all the so-called abortive methods, says of whitlow: "My own practice is, the moment I see a case of whitlow, and am sure of the diagnosis, to plunge a scalpel through all the tissues well down to the phalanx, and make as free an incision as the parts will permit. I never wait for evidence of suppuration. I am content to relieve tension, obtain local depletion, and make a way for pus in advance of suppuration. This having been done, I soak the incision for a few minutes in water as hot as can be tolerated, in order to encourage bleeding. Now is the time to apply the hot poultices without stint and without fear. I then order a brisk purgative or two, rectify any general condition that may be noted, by means of appropriate medicines, and dismiss my patient with fair assurance of speedy restoration to health and work."—*The Epitome.*

**Antipyretics.**—Recent investigations prove, with rational degree of certainty, that this class of remedies are of two-fold action in their powers of reducing high temperatures; that is, some act directly upon the heat-producing area, diminishing the amount lost. To the former class belong blood-letting, quinia and antifebrin, most prominently; to the latter, alcohol and antipyrin, while cold water has the double advantage of diminishing the production and increasing the loss.

It is firmly believed that antipyretics, in their proper application, not only act as placebos in rendering the patient more comfortable, but also as directly curative agents, notwithstanding it can be proved that the period of the disease is shortened thereby.—*Texas Courier-Record of Medicine.*

**Neutral Hydrochloride of Quinine.**—M. Clermont (*Bull Gen. de Therap*) agrees with M. Boymond as to the advantages of the basic hydrochloride of quinine, which is soluble in 21.4 times its weight of water, but recommends the neutral hydrochloride as still better fitted for hypodermic use, since it is soluble in its own weight of water and has no caustic action. It may be made either by adding a solution of chloride of barium to a solution of neutral sulphate of quinine, and separating the precipitate of barium by filtering ; or by adding hydrochloric acid to a solution of the basic hydrochloride.—*The American Practitioner and News.*

**Bromide of Potassium in Ptyalism of Pregnancy.**—In a very stubborn case of salivation during pregnancy Dr. Schramm made a trial of numerous drugs recommended for the treatment of this trouble. Iodide of potassium had no effect. Atrophine, so highly recommended by Heidenheim and Ebstein, ameliorated the condition, but had to be abandoned on account of manifestations of poisoning. Galvanization of the cervical sympathetic and subcutaneous injections of pilocarpine acted only as palliatives. Permanent cure was gained only after the use of bromide of potassium, ten grains three times a day.—*Memorabillien—Amer. Prac.*

**Antipyrin in Bilious Headache.**—John Ogilvy, M. D., writes to the *British Medical Journal*, July 16, that he has found antipyrin wonderfully potent in "bilious headache." He gives eight grains, the patient being at rest in a darkened room. At the end of an hour another dose, possibly a third, or fourth, may be required, but generally sleep, or a pleasant langour, follows the first or second dose, with gradual relief to the headache. No unpleasant after-effect is felt. He recommends a dose of the drug to be taken as a prophylactic, by those about to subject themselves to conditions which usually, in them, produce headache.—*New York Medical Abstract.*

**Treatment of Acute Miliary Tuberculosis by Iodide of Sodium.**—(Lepine, in *La Semaine Medicale.*)—It cannot be denied that cases of undoubted acute tuberculosis have been cured. The remedies that have given the best results are the preparations of iodine; iodide of sodium is the best of these. Lepine reports a case treated with iodide of sodium (ten to fifteen grains daily), which terminated fatally ; but, at the autopsy, no fresh tubercles were found—a circumstance upon which he lays great stress and which he attributes to the action of the liberated iodine.—*Deutsche Medizinal-Zeitung.*

